# “Let me handle this myself”: The value of psychological empowerment and kaleidoscope career orientation for career sustainability

**DOI:** 10.1097/HMR.0000000000000490

**Published:** 2026-04-15

**Authors:** Denise Mary Jepsen, Beatrice Van der Heijden, Sait Gürbüz

**Affiliations:** Denise Mary Jepsen, PhD, is an organizational psychologist and Professor of Management, Macquarie Business School, and the Lifespan Health & Wellbeing Research Centre, Macquarie University, Sydney, NSW, Australia; Beatrice Van der Heijden, PhD, is Full Professor and Head of the Department Strategic HRM, Institute for Management Research, Radboud University, Nijmegen, The Netherlands; Faculty of Management, Open Universiteit, Heerlen, The Netherlands; Department of Marketing, Innovation and Organisation, Ghent University, Ghent, Belgium; University of the Free State Business School, Bloemfontein, South Africa; Kingston Business School, Kingston University, London, UK. E-mail: beatrice.vanderheijden@ru.nl; Sait Gürbüz, PhD, is Professor of HRM, Organizational Behavior, and Research Methods, Positive Organizational Behavior Research Group, International Business School, Hanze University of Applied Sciences, Groningen, The Netherlands

**Keywords:** authenticity, balance, career sustainability, challenge, job satisfaction, kaleidoscope career model, psychological distress, psychological empowerment, residential aged care workers, self-determination theory, task performance

## Abstract

**Background:**

High staff turnover in residential aged care threatens both care quality and workforce sustainability, highlighting the urgent need to understand factors that support employee well-being and performance.

**Purpose:**

Building on self-determination theory and the kaleidoscope career model, this study examines how psychological empowerment, authenticity, balance, and challenge predict career sustainability among eldercare staff.

**Methodology:**

Cross-sectional survey data from 370 staff in 21 facilities in metropolitan and regional Australia were analyzed. Participants completed validated measures of the predictor variables, job satisfaction, psychological distress, and task performance. Hypothesized relationships were tested using structural equation modeling.

**Results:**

Psychological empowerment was positively related to job satisfaction and task performance and negatively related to psychological distress. Contrary to expectations, an authentic career orientation was negatively associated with job satisfaction and positively associated with psychological distress. Balanced career orientation was not associated with any sustainable career indicators. Challenge career orientation was positively associated with job satisfaction and negatively associated with psychological distress.

**Practice Implications:**

Given the predictive value of psychological empowerment and challenge career orientation for workers’ career sustainability, HR professionals and line managers need to be aware of the importance of fostering psychological empowerment in the workplace, and enabling opportunities for growth and development across the working life. In doing so, career sustainability can be protected and supported, which will be reflected in improved retention-relevant outcomes, well-being, and performance. Findings suggest that authenticity-oriented career priorities may be more difficult to realize in structurally constrained care settings.

In a rapidly evolving world of work and with increasing demands in health care settings, understanding the factors that contribute to sustainable careers in this sector has become increasingly important (e.g., Krzyzaniak et al., 2021). This issue is particularly acute in residential aged care, where chronic workforce shortages, high turnover, and persistent job strain threaten not only employees’ careers but also the continuity and quality of care delivery (Xiao et al., 2021). Despite widespread workforce concerns, there remains limited evidence explaining how health care workers’ turnover and retention can be addressed in high-demand health care settings, including residential aged care. Rather than focusing solely on job-level or organizational explanations for workforce instability, this study adopts a careers perspective to examine turnover and retention as outcomes unfolding over time.

Guided by the sustainable career framework (De Vos et al., 2020; Van der Heijden & De Vos, 2015) and its associated Process Model of Sustainable Careers (ibid) that highlights how careers are shaped by ongoing interactions between the person, context, and time dimensions, this study focuses on two individually agentic resources: psychological empowerment and kaleidoscope career orientation. At a theoretical level, the sustainable career perspective aligns closely with self-determination theory (SDT; Deci & Ryan, 2000) in its emphasis on individual agency and the psychological conditions that enable people to sustain motivation, well-being, and effective functioning across their careers. Consistent with this perspective, both kaleidoscope career orientation and psychological empowerment reflect agentic resources through which the three basic psychological needs of autonomy, competence, and relatedness are supported at work (Ryan & Deci, 2000).

A kaleidoscope career orientation is based on the kaleidoscope career model (KCM), which posits that individuals shape their careers by continuously adjusting their work to three key parameters (Mainiero & Sullivan, 2005): authenticity, balance, and challenge. These parameters reflect personal and career values and beliefs that shape an individual’s self-directed decisions and transitions, resonate with SDT’s emphasis on volition and personal growth (Deci & Ryan, 2012), and are central to how workers evaluate and adapt their careers in line with evolving life and work demands (Mainiero & Sullivan, 2005).

Psychological empowerment is a critical construct in working life and encompasses the mechanisms through which individuals gain control in organizations (Seibert et al., 2011) via meaning, competence, self-determination, and impact (Spreitzer, 1995). Employees who feel psychologically empowered are more likely to engage proactively with their work and careers in ways that promote their career sustainability, reflected in positive attitudinal and behavioral consequences in the workplace (Seibert et al., 2011).

Together, kaleidoscope career orientation and psychological empowerment represent intraindividual resources that may help individuals to foster their career sustainability over time. By examining the relationships of components reflecting these resources with the career sustainability indicators of happiness, health, and productivity (Van der Heijden, 2005), in this paper operationalized as job satisfaction, psychological distress, and task performance respectively, this study aims to advance understanding of career sustainability in the aged care workforce by examining how the authenticity, balance, and challenge dimensions of the KCM and psychological empowerment predict happiness, health, and productivity outcomes. Accordingly, this study contributes to a better understanding of employee turnover and retention from a careers perspective, highlighting how individual agentic resources support thriving in current career environments, with particular relevance for workforce sustainability in residential aged care.

## Theoretical Framework and Hypotheses

### Workforce Issues in Residential Aged Care

Residential aged care organizations provide accommodation along with personal, clinical, and everyday support for older people who can no longer live independently, with the aim of supporting their health, functioning, and quality of life. In aged care homes, residents receive assistance with routine daily activities (such as meal preparation, domestic tasks, and laundry), personal care (including bathing, dressing, and toileting), and health-related services such as medical care, nursing, and allied health. Facilities provide additional supports designed to promote social connection, emotional well-being, and meaningful engagement (Department of Health and Aged Care, 2025). Clinical and support staff work intimately with long-term resident care recipients with declining physical and mental health.

Providers of residential aged care face mounting workforce challenges, with persistent high turnover (Howe et al., 2012) and difficulties retaining qualified staff (Devi et al., 2021; Xiao et al., 2021), thereby severely threatening the quality of care provided to residents (Xiao et al., 2021). High turnover disrupts continuity of care and team cohesion and imposes significant financial and operational burdens (Tullar et al., 2016). Aged care sector–specific meta-analyses confirm that individual, organizational, and psychological factors impact employee retention (O’Keefe et al., 2025b; Thwaites et al., 2023; Wang et al., 2024). At the individual employee level, personal satisfaction and positive feelings of identity with their work roles are important retention factors, while disempowerment due to limited influence on care decisions contributes to people wanting to leave their job (Thwaites et al., 2023). At the organizational level, managers who value, recognize, and support their staff (O’Keefe et al., 2025b) and organizations with value-driven and empowered care practice (O’Keefe et al., 2025a) can enhance retention and reduce turnover of personal care workers. Psychological factors such as work stress, burnout, happiness, and job satisfaction contribute to turnover intentions (Wang et al., 2024). Employee age has mixed research results, sometimes predicting turnover intention (Wang et al., 2024) and sometimes not (Radford et al., 2015).

### Sustainable Careers and the Kaleidoscope Career Model (KCM)

Persistent employee turnover and retention challenges in aged care highlight the importance of a careers lens that conceptualizes employment as a long-term trajectory (Baruch & Sullivan, 2022). Sustainable Careers theory is a contemporary approach to career development that emphasizes balancing personal life and career goals with employer objectives (Van der Heijden, 2005). Introducing the Sustainable Careers framework, Van der Heijden and De Vos (2015) defined sustainable careers as “sequences of career experiences reflected through a variety of patterns of continuity over time, thereby crossing several social spaces, characterized by individual agency, herewith providing meaning to the individual” (p. 7). All careers involve sequences of career experiences, yet those sequences may not be equally sustainable, particularly in demanding environments such as residential aged care.

The Process Model of Sustainable Careers (De Vos et al., 2020) connects the personal, contextual, and temporal dimensions of careers and staff retention. The model highlights the interplay between individual agency (the Person dimension), contextual factors (the Context dimension), and time (the Time dimension) in shaping an individual worker’s career sustainability. Rather than treating careers as static sequences of roles, the process model emphasizes adaptive processes, feedback loops, and turning points through which individuals actively respond to changing personal and environmental circumstances (Talluri et al., 2025). As such, within the Person dimension, sustainable careers refer to an individual’s capacity to maintain a healthy, meaningful, and viable career (referred to as happiness, health, and productivity; Van der Heijden, 2005) over time. The model provides a dynamic framework for examining how individuals navigate their working lives in ways that preserve their happiness, health (i.e., reflecting the employee perspective), and productivity (i.e., reflecting the employer perspective; Van der Heijden, 2005). In this view, sustainability is not a fixed career outcome but a dynamic state that is continuously shaped by person–context transactions unfolding over time.

Consistent with the sustainable careers perspective, we propose the Kaleidoscope Career Model (KCM) that highlights how people make career decisions that align with their personal and career values, and navigate their career paths in response to changing personal and professional circumstances over time (Sullivan & Mainiero, 2008). The KCM emphasizes individuals’ ongoing recalibration of career priorities in response to shifting personal and contextual demands. In doing so, we adopt a whole-life perspective aimed at understanding the determinants of sustainable careers (cf. Hirschi et al., 2020; Van der Heijden et al., 2020) and career success (Tran Huy & Vu Hoang, 2025). In residential aged care, where much of the workforce is female and many are managing multiple caregiving responsibilities, the KCM’s relevance as a career orientation mindset (Keating, 2024) is particularly pronounced.

KCM identifies the three core parameters of authenticity, balance, and challenge that shift over different life and career stages as individuals prioritize to varying degrees. These changes in the three parameters’ domination shape how employees interpret their work experiences and assess the fit of their lives and careers, thereby impacting decisions to stay or to leave their organization. The first KCM parameter of *authenticity* involves the ability to work in a way that reflects one’s core values and sense of purpose, which residential aged care workers are likely to see as important given the relational and service-oriented nature of their roles. *Balance* reflects the need to manage competing demands between one’s work and personal life, which is frequently undermined in the residential aged care sector by inflexible rosters, short staffing, or excessive workloads. *Challenge* refers to opportunities for stimulation, growth, and advancement that can be lacking in highly routinized or undervalued aged care work. When organizational conditions limit workers’ ability to fulfill their needs for authenticity, balance, and challenge, even psychologically empowered staff may experience dissatisfaction or disconnection from their roles, risking their career sustainability.

### Psychological Empowerment

Beyond career orientation that reflects individuals’ evolving priorities, psychological empowerment captures a conceptually complementary but analytically distinct resource—the extent to which the work context enables staff to enact agency in their roles. From a self-determination perspective (Deci & Ryan, 2000), psychological empowerment reflects the extent to which work contexts satisfy employees’ basic psychological needs for autonomy, competence, and relatedness, thereby supporting sustained motivation, well-being, and effective functioning over time. Psychological empowerment offers a valuable theoretical complement to KCM for understanding employee turnover and retention in this sector.

Psychological empowerment, comprising meaning, competence, self-determination, and impact (Spreitzer, 1995), has been widely validated (Oliveira et al., 2023) and is distinct from similar constructs such as self-efficacy, self-esteem, and power (Zimmerman, 1995). Research has found that the subjective, cognitive, and attitudinal processes of psychological empowerment can lead to improved motivation and sense of purpose, with particularly strong effects on job satisfaction and organizational commitment (Llorente-Alonso et al., 2024).

Psychological empowerment has been linked in health care sector studies to greater job satisfaction (Li et al., 2018), lower burnout (Boudrias et al., 2012), stronger organizational commitment (Wagner et al., 2010), and, in nursing homes specifically, organizational citizenship behavior (Ginsburg et al., 2016). One meta-analysis found empowerment to be a “critical management strategy” for nurses having a positive impact on both patient care and nurse burnout (Şenol Çelik et al., 2024, p. 189), while an empowerment training intervention in five health care organizations had a positive impact on employee engagement and work performance (Van Dorssen-Boog et al., 2021). Beyond direct patient care, psychological empowerment has been found to play a key role in knowledge sharing in health care (Wu et al., 2022, p. 2529) and is an important mediator for innovative behavior in health care (Akkoç et al., 2022). In the aged care context, strengthening employees’ sense of purpose and control over their work may buffer against the pressures that drive turnover. By investigating how psychological empowerment operates within residential aged care and identifying whether it functions as a mechanism to enhance individuals’ career sustainability, this study aims to inform practical strategies for improving staff retention and promoting a more engaged, resilient workforce.

Integrating psychological empowerment with the KCM allows for a broad and context-sensitive understanding of retention among residential aged care staff. In particular, while psychological empowerment addresses how staff experience their current roles, the KCM captures how they navigate their careers over time, balancing personal aspirations, constraints, and hindrances related to the “dual agenda” that facilitates work and family integration in the workplace (Mainiero & Sullivan, 2005, p. 13).

Psychological empowerment contributes to workers’ career sustainability by fostering their intrinsic motivation and positive work experiences, in particular perceived control, through enhanced perceptions of meaning, competence, self-determination, and impact (Spreitzer, 1995). The KCM enriches this perspective by recognizing that employees’ career decisions are also shaped by evolving needs for authenticity, balance, and challenge throughout their careers. When these needs are unmet, despite a sense of psychological empowerment, employees may still experience strain or disengagement. Building on the core assumptions of Self-Determination theory (Deci & Ryan, 2012) and adopting a Sustainable Career theorizing lens, we integrate both perspectives and contend that both psychological empowerment and KCM offer a holistic or whole-life approach (Hirschi et al., 2020; Van der Heijden et al., 2020) to understand how residential aged care workers navigate their careers.

We suggest that psychological empowerment as an internal motivating force fosters job satisfaction (e.g., Li et al., 2018), that psychological empowerment acts as a mechanism that increases a sense of control and purpose, and therefore protects against psychological distress (e.g., Partlak Günüşen et al., 2022), and that empowered individuals are more likely to build up competencies that are needed to portray good task performance (Kundu et al., 2019). Based on this, we hypothesize:

Hypothesis 1. Psychological empowerment is associated positively with job satisfaction (a), negatively with psychological distress (b), and positively with task performance (c).

With respect to the first KCM parameter of authenticity, and focusing on being true to oneself (Simmons et al., 2022), we propose that individuals who work in ways that align with their core values experience greater job satisfaction (Metin et al., 2016), that living and working authentically decreases psychological distress (Boyraz et al., 2014) and that when people feel authentic at work, they are better able to perform well in their tasks (van den Bosch & Taris, 2014):

Hypothesis 2. Authenticity career orientation is associated positively with job satisfaction (a), negatively with psychological distress (b), and positively with task performance (c).

Regarding the second KCM parameter of balance, we contend that people putting this value to the forefront are more likely to connect more strongly to their family, friends, and others in their proximal life sphere (Simmons et al., 2022). As a result, these closer relationships are expected to reduce psychological distress. We do not expect balance to predict either job satisfaction or task performance, as it mainly focuses on developing sound social networks and providing a sense of connection to those around them, thereby assisting them in the achievement of better work/nonwork balance:

Hypothesis 3. Balanced career orientation is negatively associated with psychological distress.

Finally, with respect to the third KCM parameter of challenge that exhibits the strongest focus on work (Simmons et al., 2022), we posit that seeking and meeting challenges enhances employee engagement to invest more effort in their working life and to increase their visibility at the workplace (Gürbüz, Bakker, et al, 2026), thereby enabling them to have access to important information and resources. In doing so, we expect them to experience a high amount of job satisfaction, less psychological distress, and their intrinsic motivation to contribute beyond their role is reflected in enhanced task performance. Based on this, we hypothesize:

Hypothesis 4. Challenge career orientation is associated positively with job satisfaction (a), negatively with psychological distress (b), and positively with task performance.

## Methods

After Human Research Ethics Committee approval and written informed consent, data were collected from employees of an aged care provider in New South Wales, Australia, with a total of 1,644 employees in 21 residential villages. Villages had 31–180 staff (average 72). To provide safe, supportive, and person-centered accommodation and care, each village typically includes a general manager overseeing operations, registered nurses and personal care workers delivering clinical and personal care, and support staff such as kitchen, cleaning, laundry, and administrative personnel. Larger villages had department managers who supervised care, lifestyle, catering, and other services.

A total of 477 responses were received, for a 29% response rate. The percentage of missing values for individual items ranged from 0.3% to 3.5%. Given the minimal levels of missing data at the item level, which are unlikely to have a significant impact on the analysis, we applied the expectation-maximization algorithm to handle the missing values, as it provides unbiased parameter estimates (Newman, 2014).

### Measures

#### Psychological Empowerment

We assessed psychological empowerment using Spreitzer’s (1995) 12-item scale, which captures four facets of empowerment: meaning, impact, competence, and self-determination. An example item is “My job activities are personally meaningful to me.” Participants rated their agreement on a 7-point scale, indicating the extent to which each statement characterized them at work (1=*Extremely uncharacteristic*, 7=*Extremely characteristic*). Items were averaged to create a unidimensional composite score, with higher values indicating higher psychological empowerment. This approach was adopted to ensure a more parsimonious representation of the construct in the analyses.

#### Kaleidoscope Career Orientation

To reduce participant burden and improve response rates, we used the nine highest-loading items from the original 15-item Kaleidoscope Career Orientation scale, as identified in the original validation study by Sullivan et al. (2009). This approach allowed us to retain items that best represented the KCM dimensions while maintaining brevity. This scale captures career orientation through three parameters of authenticity, balance, and challenge, with each assessed by three items. An example item is “I hope to find a greater purpose to my life that suits who I am.” Items were rated on a 5-point scale (1=*Not at all*, 5=*Very well*). Item responses were averaged to compute composite scores for each dimension, with higher scores reflecting stronger endorsement of the respective career orientation.

#### Sustainable Career Indicators

To assess *h* *appiness*, we used the 6-item job satisfaction scale (Cammann et al., 1983). Participants were asked to describe their current job satisfaction, responding to statements such as “I really enjoy my work” on a 5-point scale (1=*Strongly disagree*, 5=*Strongly agree*). Items were averaged to create a composite job satisfaction score, with higher values indicating greater job satisfaction.

#### Psychological Distress

To assess *health*, we used the 10-item psychological distress scale (Kessler et al., 2002). Participants were asked to indicate how often they had experienced certain feelings in the past 30 days, such as being “depressed,” “nervous,” and “worthless,” using a 5-point rating scale (1=*None of the time*, 5=*All of the time*). Item responses were averaged to compute an overall psychological distress score, with higher scores indicating greater distress.

#### Task Performance

To measure *productivit* *y*, we used the 6-item (Greenhaus et al., 1990) task performance scale. An example item is “My quantity of work is higher than average.” Participants rated their agreement with statements on their work performance using a 5-point rating scale (1=*Strongly disagree*, 5=*Strongly agree*). Items were averaged to form a composite task performance score, with higher scores reflecting higher perceived performance.

#### Controls

We considered several potentially relevant control variables, including age, gender, and organizational tenure. Prior career research suggests that age is associated with career outcomes because it reflects career stage and accumulated experience, shaping both objective and subjective career outcomes (Ng et al., 2005). Gender has been widely examined due to persistent differences in career opportunities and advancement across organizational contexts, which may arise from structural and social mechanisms such as role expectations and differential access to developmental opportunities (Ng et al., 2005). Organizational tenure is often included because it reflects firm-specific human capital and embeddedness, potentially influencing access to internal labor markets and promotion opportunities (Ng & Feldman, 2008).

Given these relationships, it is possible that the focal predictor relates to the outcome not because of the theoretical mechanisms proposed in this study, but rather due to shared variance with these demographic characteristics. Accordingly, we first examined bivariate correlations between the control variables and the study constructs (Table 1). This examination indicated that age and gender were not meaningfully related to the outcome variables, whereas organizational tenure showed a modest association consistent with prior career research.

**Table 1:** Means, standard deviations, intercorrelations, and scale reliabilities (n=370)

Variables	Mean	*SD*	1	2	3	4	5	6	7	8	9
1. Psychological empowerment	5.70	0.89	.*91*	—							
2. Authenticity career orientation	3.34	0.99	.09	.*69*							
3. Balanced career orientation	4.05	0.90	.16**	.31**	.*78*						
4. Challenge career orientation	3.91	0.82	.43**	.35**	.36**	.*87*					
5. Job satisfaction	3.93	0.70	.43**	.01	.07	.32**	.*80*				
6. Psychological distress	1.82	0.94	−.29**	.23**	.03	−.18**	−.33**	.*96*			
7. Task performance	4.07	0.60	.46**	.09	.11*	.26**	.30**	−.11*	.*87*		
8. Tenure	6.04	5.60	.05	−.12*	.02	−.12*	.06	.01	.02	—	
9. Gender	—	—	−.06	−.08	−.03	.01	.01	−.01	−.06	.06	—
10. Age	44.58	13.42	.14*	−.15**	.00	.00	.01	−.19**	.10	.45**	.04

*Note.* *p<.05. **p<.01 (two-tailed). SD=standard deviation; Values in italics are Cronbach's α reliability estimates. Gender was coded as a binary variable.

We then compared structural equation models (*SEM*) estimated with and without the proposed control variables to assess the robustness of the hypothesized relationships. Although the direction and relative magnitude of the hypothesized path estimates remained substantively unchanged, the inclusion of control variables was associated with poorer overall model fit, particularly across incremental fit indices (χ^2^_[957]_=2594.16, *p*<.001; CFI=0.86; SRMR=0.10; RMSEA=0.07). In line with recommendations cautioning against the routine inclusion of control variables when construct-specific theoretical justification is limited and when their inclusion does not substantially improve explanatory value (Becker et al., 2016; Bernerth & Aguinis, 2016), we report results from the more parsimonious model without control variables to enhance interpretability and preserve statistical power.

### Analytic Approach

We used IBM SPSS 28 to conduct descriptive statistics and correlation analyses, while AMOS 28 (Arbuckle, 2021) was used to conduct confirmatory factor analyses (CFA) and structural equation modeling (*SEM*), with maximum likelihood estimation. To assess the distinctiveness of our study’s constructs, we compared the hypothesized seven-factor model (i.e., psychological empowerment, authenticity, balance, challenge, job satisfaction, psychological distress, and task performance) with several nested models: a five-factor model that merged authenticity, balance, and challenge into one factor; a three-factor model combining the three sustainable career indicators (i.e., job satisfaction, psychological distress, and task performance) into one factor; and a single-factor model that combined all items into one factor.

Model fit was evaluated using multiple indices, including χ^2^/*df*, RMSEA, CFI, and SRMR (Kline, 2023), with acceptable fit defined as χ^2^/*df* <5, RMSEA <0.08, CFI >0.90, and SRMR <0.08 (Gürbüz, 2024). The CFA results indicated that the proposed seven-factor model provided the best fit to the data (χ^2^_[831]_=1,937.76, *p*<.001; CFI=0.90; SRMR=0.07; RMSEA=0.06) compared to the alternative models (Table 2). Given that the alternative models did not outperform the proposed seven-factor model, we accepted it as the best fit and concluded that our scales are empirically distinct. Furthermore, although our study utilized a cross-sectional design, the poor fit of the single-factor model to the data provides evidence that common-method bias is unlikely to be a major concern in this study (Podsakoff et al., 2024).

**Table 2:** Comparison of hypothesized and alternative models (*n*=370)

Models	χ^2^	*df*	CFI	SRMR	RMSEA	Models	∆χ^2^	∆*df*
1. Proposed seven-factor model (PE+AU+BA+CH+JS+PD+TP)	1,937.76*	831	0.90	0.07	0.06	—	—	—
2. Five-factor model (AU, BA, and CH combined)	2,379.21*	842	0.86	0.09	0.07	2 vs. 1	441.45*	11
3. Three-factor model (JS, PD, and TP combined)	3,955.42*	849	0.72	0.11	0.10	3 vs. 1	2,017.66*	18
4. One-factor model (All items combined)	5,933.71*	852	0.54	0.15	0.13	4 vs. 1	3,955.21*	21

*Note*. *p<*.001. AU=authenticity; BA=balance; CFI=comparative fit index; CH=challenge; df=degree of freedom; JS=job satisfaction; PD=psychological distress; PE=psychological empowerment; RMSEA=Root mean square error of approximation; SRMR= standardized root mean squared residual; TP=task performance.

We used *SEM* with latent variables to test our conceptual model. *SEM* was used because it allows latent constructs to be modeled while accounting for measurement error, enables simultaneous estimation of measurement and structural components, and provides global model fit indices not available in separate regression analyses (Gürbüz, Ding, et al, 2026; Kline, 2023). Moreover, this choice is theoretically grounded in the Sustainable Career Model, which emphasizes that “sustainable career indicators should be studied in tandem in order to examine the overall sustainability of one’s career” (De Vos et al., 2020, p. 10), a requirement that *SEM* is well suited to address.

## Results

Among the final sample of 370 usable respondents, 74% were female, with a mean age of 44.6 years (*SD*=13.4), and 56.5% were employed in permanent part-time contracts. This broadly reflects the Australian aged care workforce, which is 87% female, with an average age of 47 years and 59% work permanent part-time (Department of Health and Aged Care, 2024). On average, respondents had an organizational tenure of 6 years (*SD*=5.6). Secondary education (School Certificate and Higher School Certificate) was the most common level of attainment (36.8%), followed by university degrees (Undergraduate and Postgraduate) at 33.2%, and vocational education (Certificate III, Certificate IV, and Trade or Technical courses) at 32.7%. A majority (59.7%) were married. In terms of occupational roles, 41.6% were personal care workers, while the remainder held various roles, including managers, administrative staff, coordinators, and nurses (Table 3).

**Table 3:** Respondent demographics (*n*=370)

Demographic	Category	*n* (%)	Mean	*SD*
Gender	Female	274 (74.05)		
	Male	65 (17.57)		
	Missing	31 (8.38)		
Age		326	44.58	13.42
	Missing	44 (11.93)		
Tenure		325	6.04	5.60
	Missing	45 (12.20)		
Education	School Certificate	47 (12.70)		
	Higher School Certificate	89 (24.05)		
	Trade/Tech Course or Cert 3	45 (12.16)		
	Trade/Tech Course or Cert 4	31 (8.38)		
	University—Undergraduate	89 (24.05)		
	University—Postgraduate	34 (9.19)		
	Missing	35 (9.46)		
Relationship status	Single	55 (14.86)		
	Married or de facto	221 (59.73)		
	Divorced	23 (6.22)		
	Widowed	13 (3.51)		
	Missing	58 (15.68)		
Employment type	Full-time (permanent)	83 (22.43)		
	Part-time (permanent)	209 (56.49)		
	Causal	33 (8.9)		
	Missing	45 (12.16)		
Role	Personal care workers	154 (41.62)		
	Managers	31 (8.37)		
	Administrative staff	21 (5.68)		
	Nurse	20 (5.40)		
	Coordinators	16 (4.32)		
	Others (e.g., catering officer, laundry, chef)	106 (28.64)		
	Missing	22 (5.95)		

*Note.* *SD=*standard deviation. Educational attainment was measured by allowing respondents to select all qualifications attained rather than indicating a single highest level.

Table 1 shows Pearson’s correlations, means, standard deviations, and reliabilities for all model variables. As expected, psychological empowerment was positively correlated with job satisfaction (*r*=.43, *p*<.01) and task performance (*r*=.46, *p*<.01) and negatively correlated with psychological distress (*r*=−.29, *p*<.01). Authentic career orientation was positively correlated with psychological distress (*r*=.23, *p*<.01), whereas balance was weakly positively correlated with task performance (*r*=.11, *p*<.05). Challenge career orientation was positively correlated with job satisfaction (*r*=.32, *p*<.01) and task performance (*r*=.26, *p*<.01) and negatively correlated with psychological distress (*r*=−.18, *p*<.01).

### Hypotheses’ Testing

**Figure 1:**
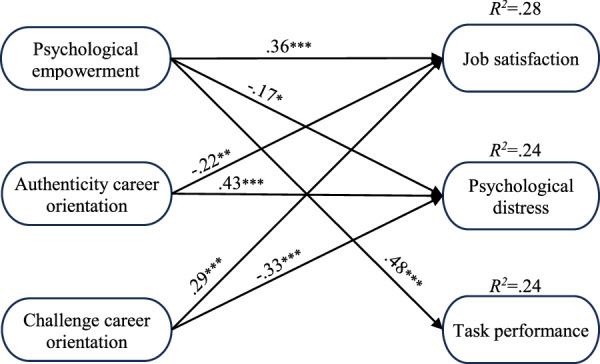
Standardized path estimates for the model of sustainable careers (*n*= 370). *Note*. *p<.05; **p<.01; ***p<.001. Only significant standardized beta estimates are shown for simplicity.

To test our research hypotheses, we performed an *SEM* analysis, specifying direct paths from the predictors (psychological empowerment, authenticity career orientation, balanced career orientation, and challenge career orientation) to the three outcome variables (job satisfaction, psychological distress, and task performance). The path model demonstrated an acceptable fit to the data (χ^2^_[834]_=1,958.90, *p*<.001; CFI=0.90; SRMR=0.07; RMSEA=0.06). Figure 1 shows significant paths.

Hypothesis 1 proposed that psychological empowerment would have a positive association with job satisfaction (a), a negative association with psychological distress (b), and a positive association with task performance (c). As expected, the *SEM* results revealed that employees with higher psychological empowerment reported more job satisfaction (β=0.36, *p*<.001), enhanced task performance (β=0.48, *p*<.001), and lower psychological distress (β=−0.17, *p*<.05). These findings support Hypotheses 1a, 1b, and 1c.

Hypothesis 2 stated that the authenticity career orientation would be positively associated with job satisfaction (a), negatively associated with psychological distress (b), and positively associated with task performance (c). Contrary to our expectations, the results showed that authenticity was negatively associated with job satisfaction (β=−0.22, *p*<.01) and positively associated with psychological distress (β=0.43, *p*<.001), contradicting Hypotheses 2a and 2b. The relationship with task performance was not significant (β=0.05, *p*=.451), failing to support Hypothesis 2c. While bivariate correlations showed no significant link between authenticity and job satisfaction (*r*=.01, *p*=.933), the *SEM* results suggest a negative association. These discrepancies led us to conduct a supplementary analysis, which we report below.

Hypothesis 3 was that balanced career orientation would be negatively associated with psychological distress. *SEM* analysis showed that balance was not significantly associated with psychological distress (β=0.04, *p*=.532), providing no support for Hypothesis 3.

Finally, Hypothesis 4 stated that challenge career orientation would be positively associated with job satisfaction (a), negatively associated with psychological distress (b), and positively associated with task performance. As expected, *SEM* analysis showed that employees who experienced higher levels of challenge were more satisfied with their job (β=0.29, *p*<.01) and reported lower psychological distress (β=−0.33, *p*<.01), thereby supporting Hypotheses 4a and 4b. However, the relationship between challenge and task performance was not significant (β=0.01, *p*=.909), providing no support for Hypothesis 4c.

### Supplementary Analysis

While bivariate correlations showed no significant link between authenticity and job satisfaction, our *SEM* results indicated a negative association. Such a discrepancy may be due to complex relationships among the predictors, such as suppression effects or shared variance with other predictors (Gürbüz, 2024). Differences between bivariate correlations and *SEM* path estimates reflect the simultaneous estimation of multiple correlated career orientation dimensions and the isolation of their unique effects, rather than multicollinearity in the regression sense. To explore this further, we conducted a supplementary analysis where we modeled an *SEM* with authenticity as the sole predictor of job satisfaction. The results of the path model (χ^2^_[270]_=768.59, *p*<.001; CFI=0.91; SRMR=0.08; RMSEA=0.07) showed a nonsignificant negative association between authenticity and job satisfaction (β=−0.08, *p*=.226), reinforcing the finding of an absence of a direct relationship between these two variables in our sample.

## Discussion

This study examined how psychological empowerment and the core dimensions of the kaleidoscope career model relate to indicators of career sustainability—job satisfaction, psychological distress, and task performance—among residential aged care workers. Overall, the findings indicate that psychological empowerment and challenge-oriented career priorities emerge as the most consistent resources supporting career sustainability, whereas authenticity-oriented priorities were associated with poorer well-being outcomes, and balance showed no protective effects. These patterns offer important insights into how individual agency operates in high-demand care settings.

Consistent with prior research in health care (Spence Laschinger et al., 2009), psychological empowerment emerged as the most robust predictor across all three indicators of career sustainability. The strong associations with both job satisfaction and task performance, alongside reduced psychological distress, reinforce evidence that empowered care workers are better able to cope with demanding work environments while maintaining engagement and effectiveness (e.g., Seibert et al., 2011). From a sustainable careers perspective, these findings underscore psychological empowerment as a critical mechanism through which agency can be enacted in high emotional and physical demands contexts.

Contrary to expectations, authenticity-oriented career priorities were associated with lower job satisfaction and higher psychological distress. While authenticity is often linked to positive career outcomes in professional and managerial samples, this finding aligns with evidence (Edwards & Cable, 2009; Kristof-Brown et al., 2005), including from care contexts (Brotheridge & Grandey, 2002), suggesting that value congruence alone may not protect well-being when workers face structural constraints, emotional labor, and limited discretion. In residential aged care, workers who place strong emphasis on authenticity may experience heightened distress when their values of care quality and relational engagement cannot be fully enacted due to time pressure, staffing shortages, or organizational constraints.

The absence of a significant association between balance orientation and psychological distress suggests that prioritizing work–life balance may be difficult to realize as a protective resource in residential aged care, where shift work, staffing shortages, and limited schedule control are common. This finding echoes prior research on flexible work–life initiatives and arrangements (Allen et al., 2012; Kossek et al., 2010), indicating that individual preferences for balance may be insufficient to mitigate strain in structurally constrained care settings, highlighting the limits of individual agency in the absence of supportive organizational conditions.

Consistent with our expectations, we found that a challenge career orientation was associated with higher job satisfaction and lower psychological distress. This finding aligns with research suggesting that challenge and opportunities for growth can enhance motivation and engagement, even in demanding work environments (Van den Broeck et al., 2008). In health care settings, where work is often emotionally and physically taxing, challenge-oriented individuals may be better equipped to derive meaning and resilience from their roles, thereby protecting their well-being (Naja et al., 2021; Xanthopoulou et al., 2007) and positively impacting their motivation and performance (Öztürk et al., 2025).

However, the absence of a significant association between challenge orientation and task performance suggests important contextual boundaries. In residential aged care, work is highly standardized and regulated, which may constrain opportunities for motivated employees to translate personal challenge into observable performance outcomes (e.g., Grant et al., 2011). This finding highlights that while challenge may support retention-relevant outcomes such as satisfaction and reduced distress, its performance benefits may be limited by the structural characteristics of care work.

### Practice Implications

The findings point to psychological empowerment as a central leverage point for workforce sustainability in residential aged care. Practices that enhance autonomy, competence, and perceived impact, such as participative decision-making, role clarity, and supportive supervision, are likely to yield benefits for retention-relevant outcomes, including job satisfaction, well-being, and performance.

Importantly, the findings caution against assuming that encouraging authenticity will necessarily enhance well-being in aged care settings. For workers with strong authenticity-oriented career priorities, organizational constraints may amplify frustration and distress when values cannot be enacted. HR practices should therefore focus not only on encouraging value alignment but also on reducing structural barriers that prevent care workers from acting in accordance with their professional and personal values, such as excessive workloads and limited discretion.

Given the absence of significant effects for balance orientation, efforts to support work–life balance in aged care are unlikely to succeed through individual-level initiatives alone. Structural interventions such as staffing adequacy and predictable scheduling may be required before balance-oriented preferences can translate into improved outcomes.

The positive associations between challenge orientation and job satisfaction and lower distress suggest that clear development pathways, role enrichment, and opportunities for skill development may be particularly important for retaining challenge-oriented staff, even where promotion opportunities are limited. Career ladders are recommended to support those staff specifically seeking challenge, yet will also support those seeking a long, authentic career with increasing responsibility, and those with increasing financial needs seeking balance in their careers. Given the idiosyncrasy of personal lives and careers (Van der Heijden, 2005), specific bundles of HRM practices that focus on the maintenance or development of employee careers will be beneficial (Veth et al., 2019).

### Limitations and Future Research

Our sample included residential aged care workers from a single provider in New South Wales, Australia. More research across different countries and occupational categories within the aged care sector, and different types of employment such as residential and home care, is needed to more safely conclude whether these results are generalizable in a broader sense. In addition, as career sustainability is not a fixed process, longitudinal research is needed to provide important insights into how career (un)sustainability resulting from experiences in both one’s private life and working life might change or modify itself over time. Longitudinal research with multiple waves is also needed to investigate how the impact of career shocks unfolds over time, and relationships between antecedents and outcomes of this phenomenon. Moreover, although employment type and occupational role may influence career experiences and well-being, these factors were beyond the scope of the present study. Future research could explore how the relationships observed here differ across roles or employment arrangements, particularly in heterogeneous occupational contexts. Last, we call for further research on those in the aged care sector with a higher authentic career orientation to identify the obstacles to increased job satisfaction and reduced psychological distress that that orientation would predict. We hope to inspire others to help increase the career sustainability of health care professionals.

Given the severe staff shortages in the health care sector, it is crucial for management to prioritize career sustainability. When management’s focus on performance (i.e., productivity component of sustainable careers) and workers’ focus on well-being (i.e., happiness and health components) are aligned, both individuals and organizations can thrive, enhancing the likelihood of a long-term, high-quality social exchange relationship (Fugate et al., 2021).

This study demonstrates the value of adopting a careers perspective to understand turnover and retention in high-demand health care settings. Among residential aged care workers, psychological empowerment and challenge-oriented career priorities emerged as the most consistent resources associated with career sustainability, while authenticity and balance appeared more contingent on contextual conditions. These findings highlight the importance of aligning workforce strategies not only with organizational performance goals but also with the ways employees experience agency and opportunity over time. By integrating sustainable careers theory with evidence from health care, the study contributes to ongoing efforts to design retention strategies that support a stable, resilient, and sustainable care workforce.
